# Single-cell analysis and machine learning identify psoriasis-associated CD8^+^ T cells serve as biomarker for psoriasis

**DOI:** 10.3389/fgene.2024.1387875

**Published:** 2024-06-10

**Authors:** Sijia He, Lyuye Liu, Xiaoyan Long, Man Ge, Menghan Cai, Junling Zhang

**Affiliations:** ^1^ Graduate School of Tianjin Medical University, Tianjin, China; ^2^ Graduate School of Tianjin University of Traditional Chinese Medicine, Tianjin, China; ^3^ The Second Affiliated Hospital of Guizhou Medical University, Kaili, Guizhou, China; ^4^ Tianjin Academy of Traditional Chinese Medicine Affiliated Hospital, Tianjin, China

**Keywords:** single-cell RNA sequencing, hdWGCNA, machine learning, psoriasis, CD8^+^ T cells, predictive modeling

## Abstract

Psoriasis is a chronic inflammatory skin disease, the etiology of which has not been fully elucidated, in which CD8^+^ T cells play an important role in the pathogenesis of psoriasis. However, there is a lack of in-depth studies on the molecular characterization of different CD8^+^ T cell subtypes and their role in the pathogenesis of psoriasis. This study aims to further expound the pathogenesy of psoriasis at the single-cell level and to explore new ideas for clinical diagnosis and new therapeutic targets. Our study identified a unique subpopulation of CD8^+^ T cells highly infiltrated in psoriasis lesions. Subsequently, we analyzed the hub genes of the psoriasis-specific CD8^+^ T cell subpopulation using hdWGCNA and constructed a machine-learning prediction model, which demonstrated good efficacy. The model interpretation showed the influence of each independent variable in the model decision. Finally, we deployed the machine learning model to an online website to facilitate its clinical transformation.

## 1 Introduction

Psoriasis is a usual chronic inflammatory skin disease with scaly erythema or plaques as the main clinical manifestations, long duration and easy to recur (Rapalli et al., 2020). The global prevalence of psoriasis is about 3% and is increasing year by year (Scher et al., 2019). Currently, the diagnosis of psoriasis is mainly based on the characteristics of skin lesions and skin imaging, while histopathology is needed to assist in the diagnosis of some atypical lesions, but due to the traumatizing nature of biopsy, more portable auxiliary diagnostic methods are needed in clinical working. At present, psoriasis is still incurable. Mild psoriasis is mainly treated with topical therapy, while moderate to severe psoriasis needs to be treated with combined systemic medication, and patients who have poor response to traditional therapeutic drugs need to choose biological agents or small molecule drug therapy. The pathogenesis of psoriasis is complex, and it is currently believed that it is caused by immune abnormalities induced by genetic and environmental factors (Liu et al., 2024), in which a variety of cells and the cytokines they release play an considerable role in the pathogenesy of psoriasis (Hawkes et al., 2017), but the current treatment methods are only aimed at some of the links, and thus have limited efficacy, and an in-depth understanding of the cellular and molecular pathogenesis of psoriasis can help to find new therapeutic targets to optimize the combination of treatment options. This is of great clinical significance.

Through the application of single-cell sequencing technology, we are granted the opportunity to delve deeply into the immunological microenvironment within psoriatic tissues. Meticulous analyses of cellular components in both diseased and normal tissues have revealed specific subpopulations exhibiting heightened expression within psoriatic tissues. Through cell communication analyses, we have unveiled the molecular mechanisms through which these subpopulations exert their influence within the tissue microenvironment, offering a novel perspective for a nuanced comprehension of the finely tuned dynamics of the immune system during disease progression. This not only provides unprecedented opportunities for personalized therapies but also establishes the groundwork for more precise interventions.

**FIGURE 1:**
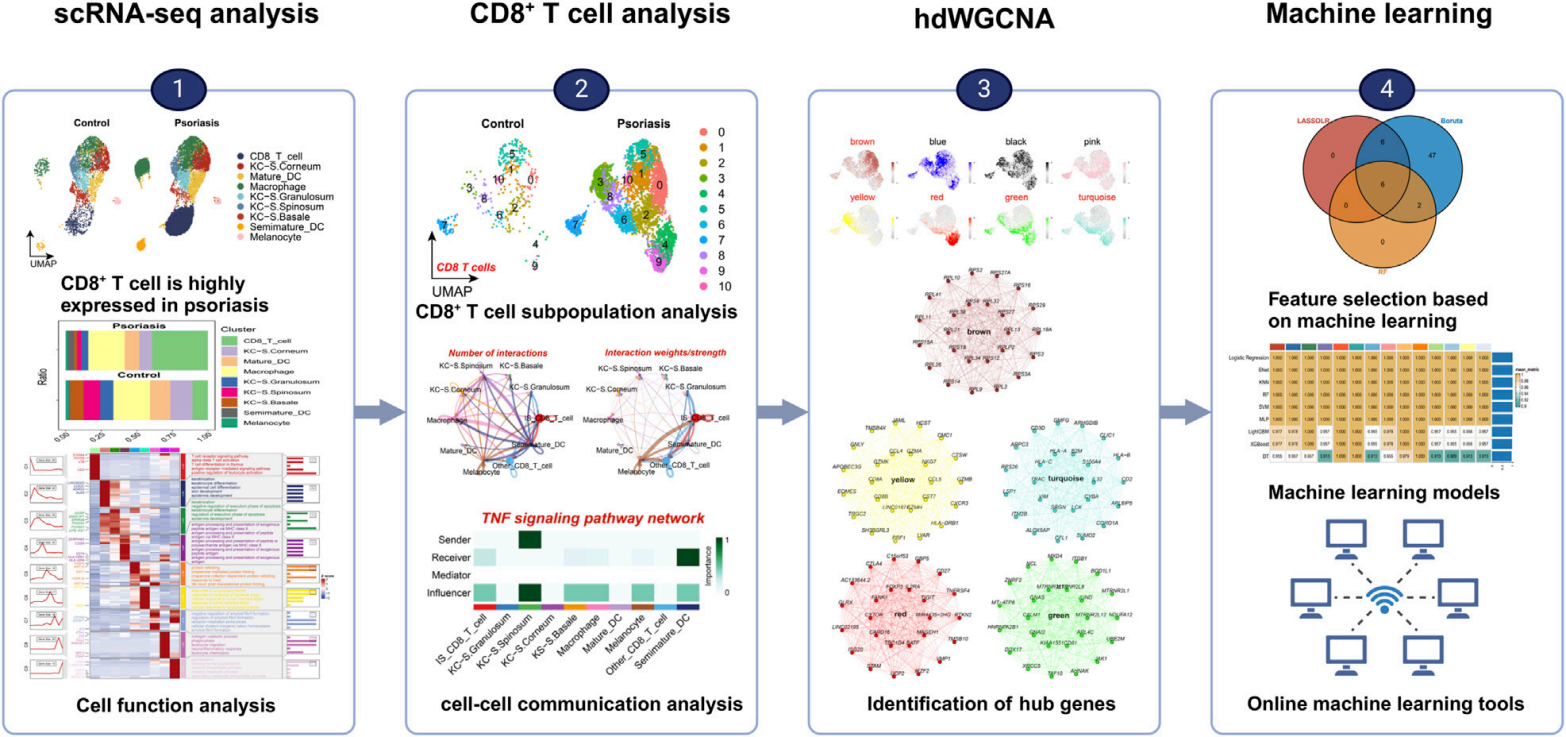
The flow chart of this study.

Ultimately, we introduce the power of machine learning, employing various algorithms to construct a diagnostic model for psoriasis based on single-cell-level information within bulk RNA sequencing (The flowchart is shown in Figure 1). This model not only accurately distinguishes psoriasis patients but also serves as an intelligent tool for clinicians (https://hesijia.shinyapps.io/Psoriasis/), facilitating early diagnosis and more personalized treatment approaches. This comprehensive research methodology opens new avenues for in-depth exploration of psoriasis, contributing positively to the enhancement of patients’ quality of life and treatment outcomes.

## 2 Materials and methods

### 2.1 Data acquisition

We downloaded the scRNA-seq dataset GSE151177 (13 human psoriasis skin and 5 healthy volunteer skin) from the Gene Expression Omnibus (GEO) database (https://www.ncbi.nlm.nih.gov/geo/) (Kim et al., 2021). Bulk RNA-seq dataset GSE54456 (92 psoriatic and 82 normal punch biopsies) were obtained from the GEO database (Li et al., 2014).

### 2.2 scRNA-seq data analysis

R package Seurat was utilized for conducting the scRNA-seq analysis. Cell clustering was implemented by the “FindNeighbors” and “FindCluster” functions of the Seurat package. We manually annotated cell types using the original literature. Intercellular communication networks with annotated scRNA-seq data were analyzed using the R package CellChat and examined for ligand-receptor-mediated interplays between diverse cell types. Assessing major signal inputs and outputs in cell subpopulations by CellChatDB.Human on cellchat. The functional enrichment analysis of cell types was performed by R package clusterProfiler.

### 2.3 hdWGCNA analysis

The hdWGCNA workflow in R was implemented using the “hdWGCNA” package (Morabito et al., 2021; Morabito et al., 2023). Briefly, the hdWGCNA pipeline consists of the following steps: 1) data preprocessing, 2) gene network construction, 3) module identification, 4) module preservation analysis. The first step is to preprocess the gene expression data to get rid of noise and batch effects. The next step is to construct the gene co-expression network according to the pairwise correlation between genes. The third step involves identifying modules or gene clusters of highly related genes and further calculate module genes. The fourth step is to perform a module preservation analysis to evaluate the stability of the identified modules.

### 2.4 Machine learning and deep learning

We applied univariate logistic regression to recognize key diagnostic genes in IS CD8^+^ T cells. In addition, the variables were filtered using the Least Absolute Shrinkage and Selection Operator Logistic Regression (LASSOLR) algorithm, Boruta and Random Forest (RF) (Kang et al., 2021; Younis et al., 2021). Machine learning models were developed by the tidymodels package (https://www.tidymodels.org/). Nine machine learning models were used to select the optimum model, including, Logistic Regression, Enet, k-nearest neighbor (KNN), RF, SVM, multilayer perceptron (MLP), LightGBM, XGBoost and decision tree (DT). All models were evaluated on the test data. SHapley Additive exPlanations (SHAP) was used for model interpretation (Wang et al., 2021). A convolutional neural network (CNN) based deep learning model was developed using the Keras and TensorFlow frameworks.

### 2.5 Statistical analysis

R version 4.2.2. was used for analysis. The statistical significance level was set at *p* < 0.05. Pearson correlation analysis was used to reveal the correlation between the 6 hub genes and 10 types of immune cells.

## 3 Results

### 3.1 Single-cell RNA sequencing analysis identifies CD8^+^ T cells upregulated in psoriasis skin lesions

**FIGURE 2:**
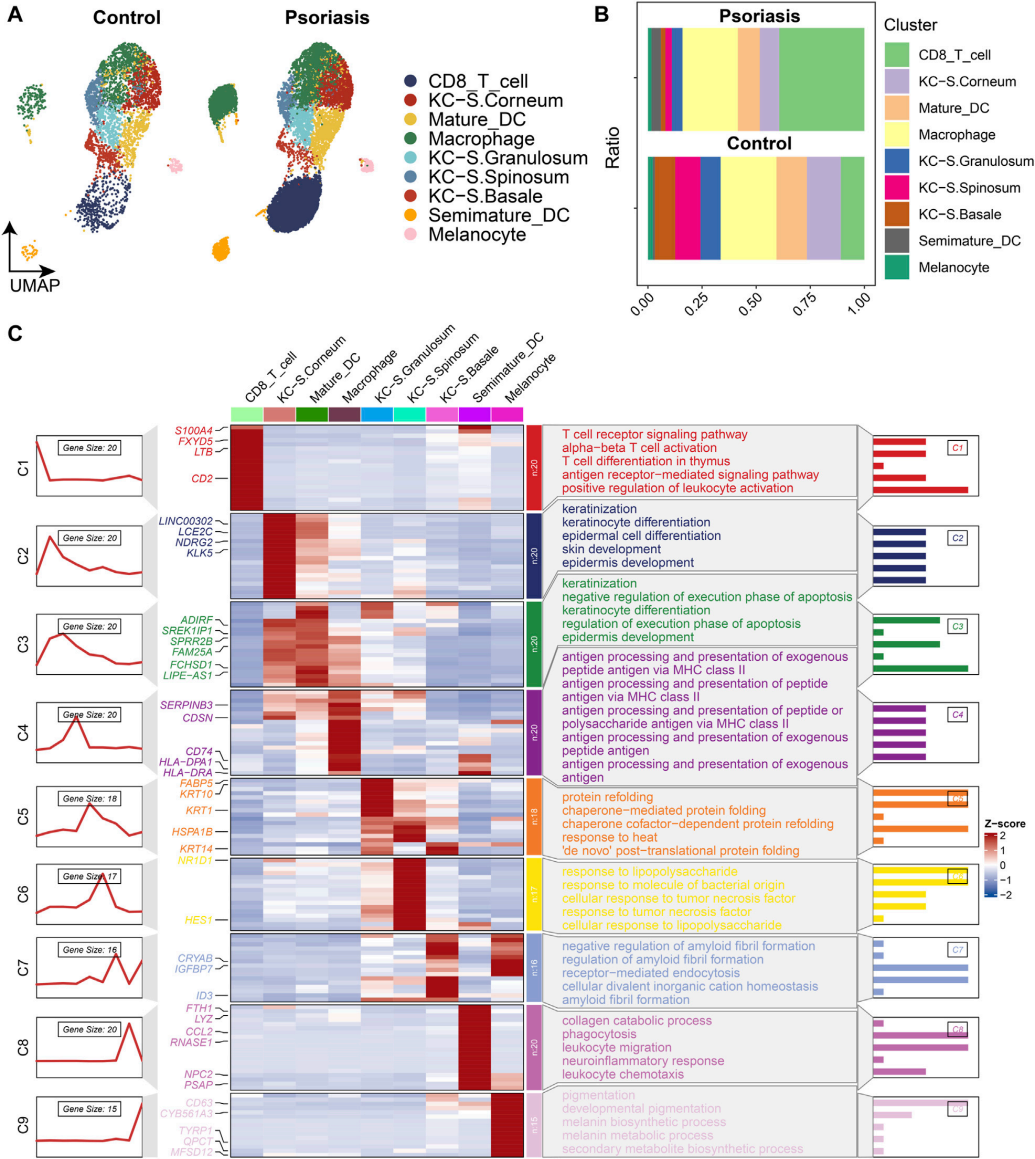
Heterogeneity of cellular composition in normal and psoriatic tissues revealed by single-cell RNA sequencing analysis according to dataset GSE151177. **(A)** UMAP scatter plot showing increased proportion of CD8^+^ T cells in psoriatic lesions. **(B)** Stacked bar graphs showing changes in the proportion of each cell type. **(C)** Functional enrichment plot showing the most significant enrichment terms for each cell type.

To explore the differences in cellular composition between psoriatic lesions and normal skin tissues, we used GSE151177 to analyze the cellular component of the two. UAMP analysis identified nine major cell populations, and after integration through harmony, dimensionality reduction, clustering and cell type annotation, we found that the alteration in the proportion of CD8^+^ T cells was most significant in psoriatic lesions (Figures 2A, B). To further understand the functions of various cell types in psoriasis lesions, we performed gene enrichment analysis, which showed that CD8^+^ T cell-related genes were mainly involved in positive regulation of leukocyte activation, T cell receptor signaling pathway, αβT cell activation and other processes (Figure 2C). Keratinocyte (KC) -related genes located in different locations of the epidermis were involved in different response processes, for example, KC-related genes located in the basal layer were mainly involved in receptor-mediated endocytosis, KC-related genes located in the stratum spinosum were mainly involved in lipopolysaccharide response, and KC-related genes located in the stratum granulosum were mainly involved in protein refolding process. With the gradual migration of KC to the stratum corneum, KC-related genes play an important role in keratinization and epidermal development. In addition, mature dendritic cell (DC) -related genes were mainly involved in epidermal development and keratinization, while semi-mature DCs mainly mediated phagocytosis and leukocyte migration. Macrophage-related genes are mainly involved in antigen processing and presentation.

### 3.2 Identification of CD8^+^ T cell subpopulations in psoriatic lesions and analysis of cellular communication

**FIGURE 3:**
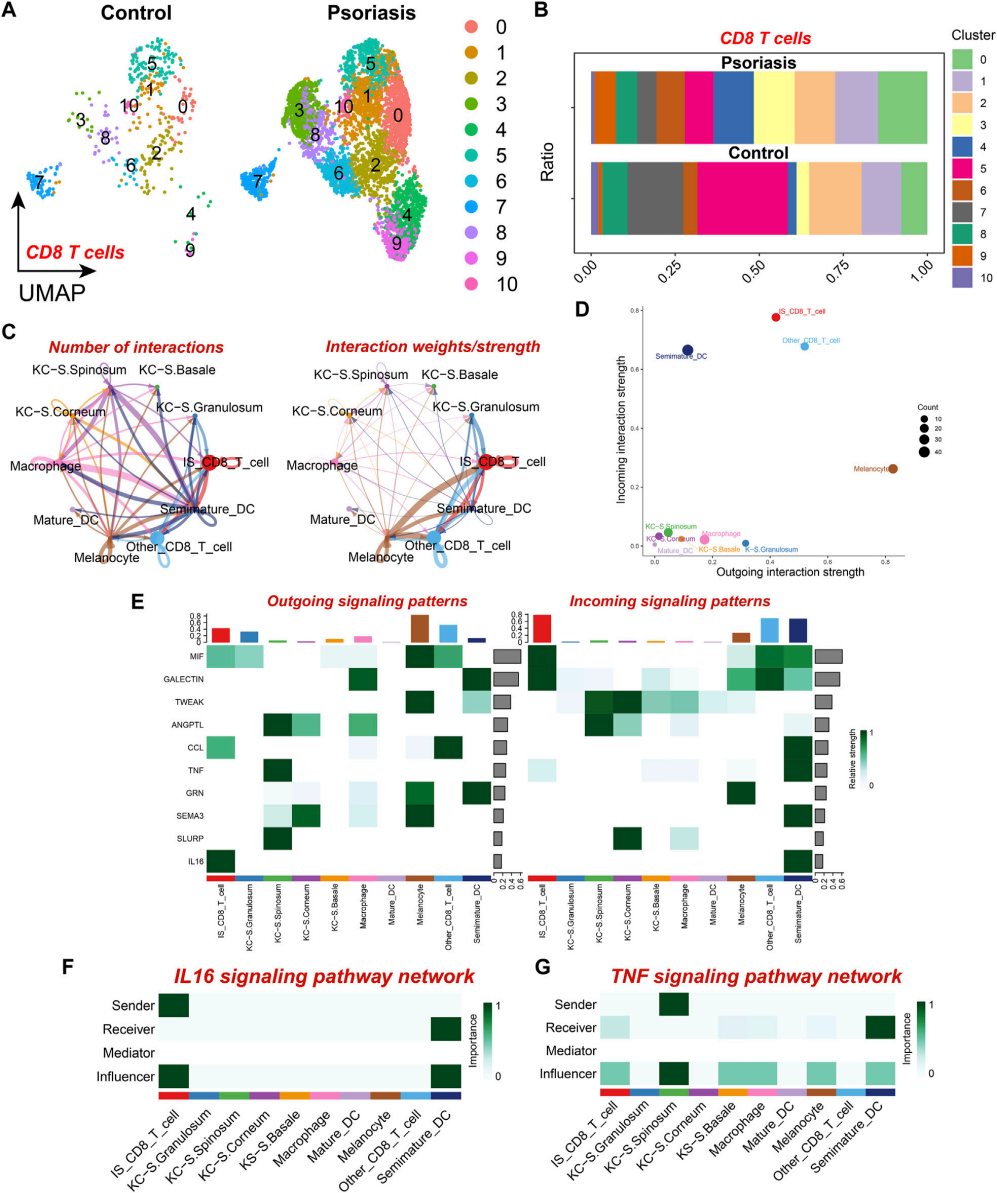
CD8^+^ T cell subset analysis and cell communication analysis **(A)** UMAP scatter plot showing the distribution of CD8^+^ T cell subtypes in psoriatic lesions and normal skin. **(B)** Stacked bar graph showing the proportion of CD8^+^ T cell subtypes in psoriatic lesions and normal skin. **(C)** The number and strength of intercellular communication between various cell subtypes. The colors of the bubbles and lines in the graph indicate different cellular sources. **(D)** Scatter plot showing the strength of input and output interactions of various cell subtypes. **(E)** Signaling role analysis on the aggregated cell-cell communication network of all signaling pathways among various cell subtypes. **(F)** Role of various cell subtypes in the IL-16 signaling pathway network. **(G)** Role of Various cell subtypes in the TNF signaling pathway network.

To further elucidate the variations in the composition of CD8^+^ T cell subpopulations in psoriatic lesions, we further identified ten different CD8^+^ T cell subsets by dimensionality reduction and clustering, which showed that not all CD8^+^ T cells were upregulated in psoriatic lesion samples, there was a significant increase in CD8^+^ T cell subsets 0, 3, 4, 6, and 9, as compared to the normal skin tissue samples (Figures 3A, B), which resulted in an elevated proportion of CD8^+^ T cells. Therefore, we selected the five subpopulations that were specifically upregulated in psoriatic lesions for further analysis and defined them as the psoriasis-specific CD8^+^ T cell subpopulation (IS CD8^+^ T cells), and defined the other CD8^+^ T cell subpopulations as the non-psoriasis-specific CD8^+^ T cell subpopulation (Other CD8^+^ T cells). To further explore the cell-cell communication network between IS CD8^+^ T cells and other cell types, we performed intercellular communication analysis and found that IS CD8^+^ T cells had even more and closer connections with other cell types, particularly with melanocytes (MC) and Other CD8^+^ T cells (Figure 3C). Moreover, among the ten cell types, MC emitted the most signals while IS CD8^+^ T cells received the most signals (Figure 3D). IS CD8^+^ T cells emitted more IL16 signals compared to Other CD8^+^ T cells and this signal was mostly received by Semimature DC (Figures 3E, F). Based on the above results, we speculated that such enhanced signaling might enhance the antigen presenting ability of semimature DC cells and the ability to activate CD4^+^ T cells, thus promoting the immune response. In addition, IS CD8^+^ T cells received more TNF signals from KC located in the stratum granulosum than Other CD8^+^ T cells (Figure 3G). From this, we inferred that specifically receiving too much TNF signaling and sending too much IL16 signaling may make IS CD8^+^ T cells a pathogenic subpopulation in psoriasis. Therefore, targeted intervention of IL16 signaling emitted by IS CD8^+^ T cells, as well as interfering with the reception of TNF signaling by IS CD8^+^ T cells, may be potential therapeutic targets for psoriasis.

### 3.3 hdWGCNA shows that IS CD8^+^ T cells are characterized by brown, yellow, green, red, and turquoise modules

**FIGURE 4:**
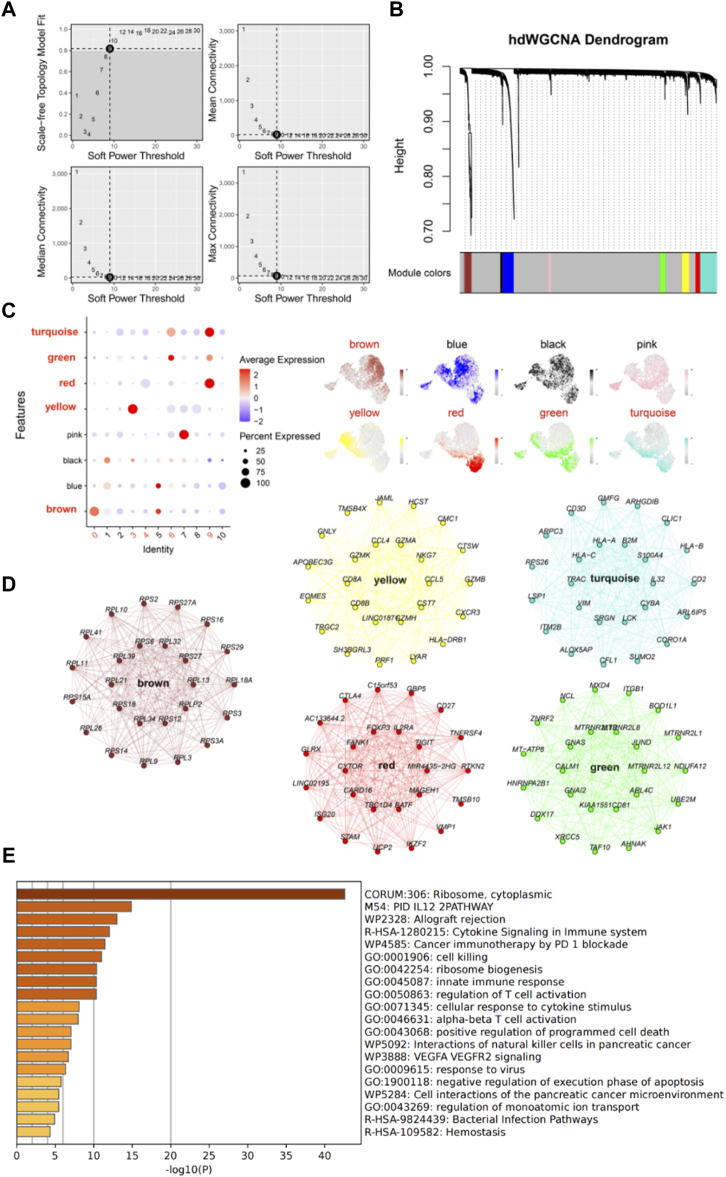
High dimensional weighted gene co-expression network analysis (hdWGCNA) further characterizes IS CD8^+^ T cells. **(A)** Power value equal to 9 when the network reached a scale-free distribution. **(B)** 8 modules were identified by hdWGCNA clustering tree. **(C)** Feather plots depict the scores of the corresponding modules in IS CD8^+^ T cells. **(D)** Network plots show the top 25 genes most associated with the five color modules. **(E)** The enrichment analysis diagram shows the most significant enrichment items of 125 genes.

We further investigated the function and characteristics of IS CD8^+^ T cells using hdWGCNA. We chose a power value of 9 to construct a scale-free network, and 8 gene modules were generated accordingly (Figures 4A, B). Among these modules, brown, yellow, green, red, and turquoise modules were highly expressed in subpopulations 0, 3, 4, 6, and 9 (Figure 4C). We then screened the top 25 genes most associated with each color module (Figure 4D), for a total of 125 genes, for use to develop machine learning diagnostic models described below. To obtain a more comprehensive understanding of these 125 genes, we performed gene enrichment analysis with Metascape (https://metascape.org/). The results showed that these genes were mainly involved in cell biosynthesis, IL12 pathway, cytokine signaling and immune response (Figure 4E).

### 3.4 Development of a machine learning diagnostic models on account of psoriasis-associated CD8^+^ T cell hub genes

**FIGURE 5:**
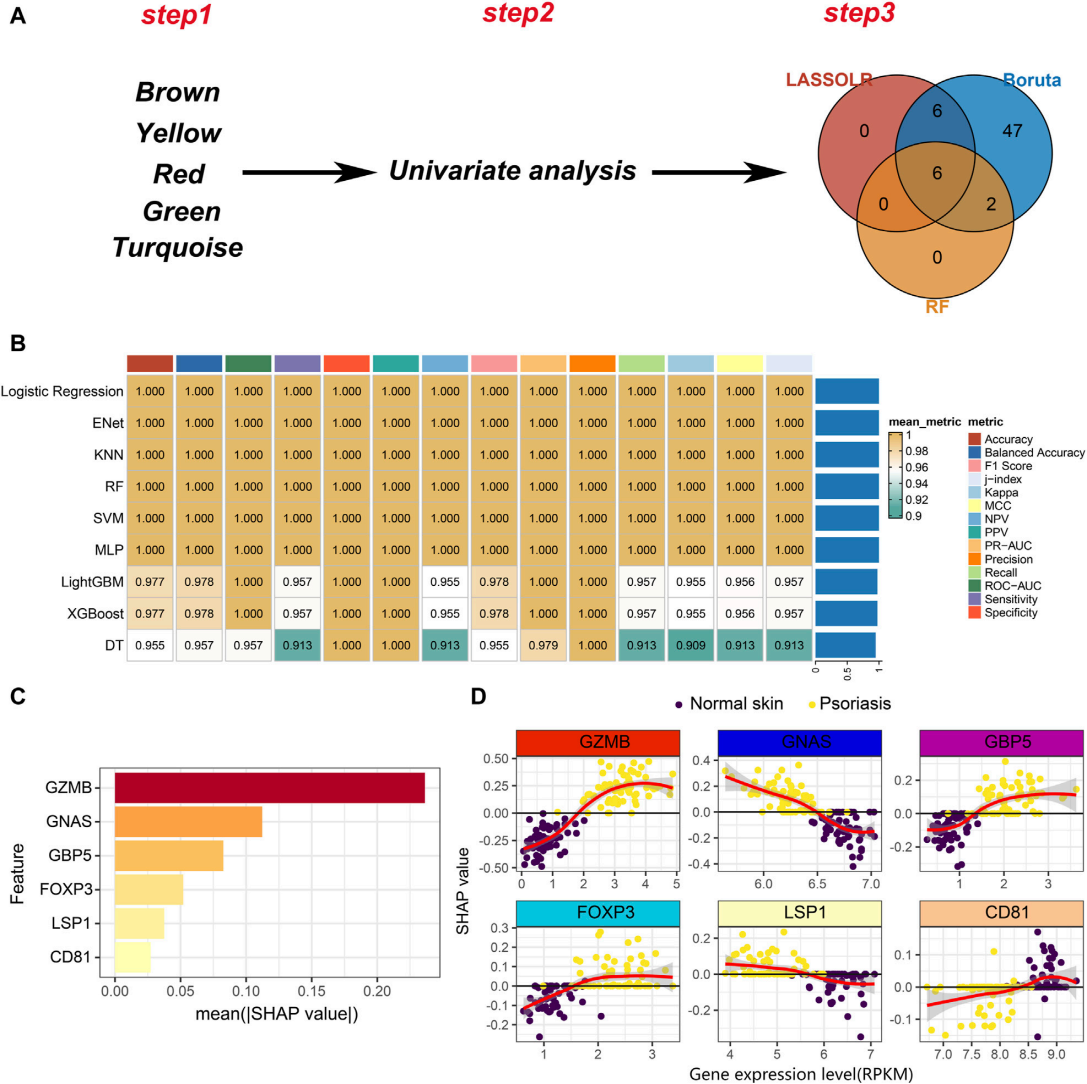
Development of machine learning models. **(A)** A three-step pipeline for hub genes selection. Step1: identification of five psoriasis-associated module genes. Step2: univariate analysis to screen the psoriasis-associated genes. Step3: Three machine learning algorithms for further selection of the psoriasis-associated genes. **(B)** Evaluation of robustness of the nine machine learning algorithms on test data. **(C)** The absolute value of the SHAP value of each feature. The higher SHAP value represents higher importance in predicting the presence of psoriasis. **(D)** Scatter plot of gene expression levels versus SHAP values for each feature in each sample.

As mentioned, we have identified five modules, including brown, yellow, red, green and turquoise. The top 25 genes in each module were selected, resulting in a total of 125 hub genes, which represent psoriasis-associated CD8^+^ T cell subsets hub genes. A total of 110 hub genes were included in the bulk RNA-seq cohort. Then, univariate analysis was performed on the 110 hub genes and 95 genes with *p* < 0.05 remained for the following analysis. Finally, three machine learning algorithms including Boruta, least absolute shrinkage and selection operator (LASSO) and RF were applied to feature selection (Figure 5A). Six features including GZMB, GNAS, GBP5, FOXP3, LSP1 and CD81 were obtained by interacting the three machine learning algorithms.

Nine machine learning algorithms were used to fit models based on the six features, including Logistic Regression, Enet, KNN, RF, SVM, multilayer perceptron (MLP), LightGBM, XGBoost and decision tree (DT). We randomly divided the bulk RNA-seq data into training set and test set in a ratio of 7:3. Nine machine learning models were trained on the training set and comprehensively evaluated on the test set (Figure 5B). We noticed that nearly all models exhibit great performance on test set. To better understand the contribution of features to model predictions, we selected the logistic regression model to conduct model explanatory analysis using SHAP. Among the six features, GZMB plays the most important role on influencing the prediction results (Figure 5C). The relationship between the expression of each feature and the SHAP value is shown in Figure 5D. A SHAP value greater than 0 means that it has a positive contribution to predicting the outcome as psoriasis. As the expression level of GZMB increases, the SHAP value gradually increases, indicating that the high expression of GZMB in CD8^+^ T cells drives the pathogenesis of psoriasis. Similarly, decreased expression level of GNAS in CD8^+^ T cells also promotes the development of psoriasis (Figure 5D). In summary, we identified 6 markers and constructed machine learning models to improve psoriasis diagnosis. Additionally, we also provided novel targets for CD8^+^ T cell-targeted therapy in psoriasis patients. For example, the use of GZMB knockout CAR-T cells in patients may delay psoriasis progression.

### 3.5 Establishment and validation of the novel convolutional neural network deep learning model

**FIGURE 6:**
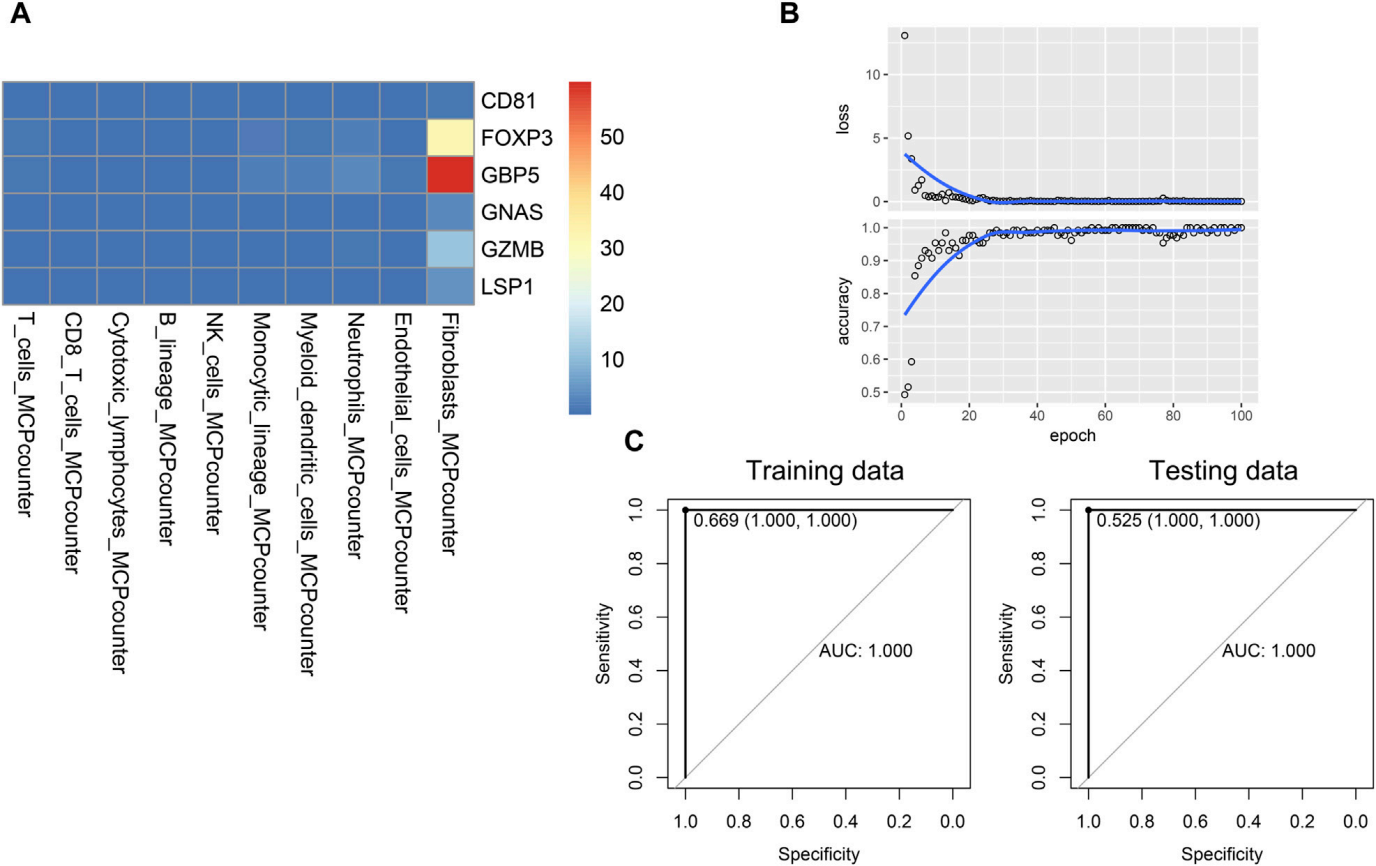
Establishment and validation of the novel CNN deep learning model. **(A)** Image of a patient used for training CNN model. **(B)** Training process of the CNN model. **(C)** Performance of the CNN model in the training and testing data.

Given that interactions exist between hub genes and other immune cells in the immune microenvironment, and these interactions may be associated with the progression of psoriasis. We developed a novel gene-immune image-based convolutional neural network (CNN) model. The advantage of the model is independent of the batch effect of different datasets. We constructed a heatmap for each patient, and the value of each square was the ratio of that patient’s gene expression to the infiltration of specific immune cells (Figure 6A). Based on the heatmaps, the CNN was trained using 100 epochs in the training set and tested in the testing set (Figure 6B). The CNN model performed well in both the training and testing sets (training AUC = 1, sensitivity = 100%, specificity = 100%, testing AUC = 1, sensitivity = 100%, specificity = 100%), suggesting good diagnostic performance (Figure 6C).

### 3.6 Development of an online web tool to deploy the machine learning model

**FIGURE 7:**
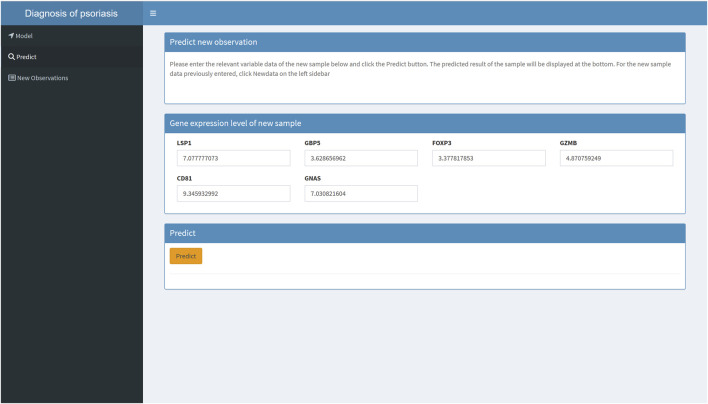
An online machine learning model for psoriasis diagnosis.

To make it easier for patients and clinicians to use the psoriasis diagnostic model we developed, we deployed the machine learning model on an online website (https://hesijia.shinyapps.io/Psoriasis/) (Figure 7).

## 4 Discussion

As a chronic inflammatory skin disease mediated by T-lymphocytes under the background of polygenic inheritance, psoriasis is stubborn and prone to recurrent episodes, and can lead to a variety of complications such as metabolic syndrome, cardiovascular disease, and arthritis (Griffiths et al., 2021), which critically affects the living quality of patients and their physical and mental health. The etiology of psoriasis has not been fully elucidated, among which genetic, environmental and immune factors play a significant role in the occurrence and development of psoriasis (Armstrong and Read, 2020). In recent years, the treatment of psoriasis with biologics has made great breakthroughs, but it still cannot meet the treatment needs of all psoriasis patients, suggesting that there are still blind spots in the underlying pathogenesis of psoriasis, so it is particularly important to gain a deeper understanding of the complex molecular mechanisms of psoriasis. In recent years, the development of single-cell RNA sequencing technology and machine learning has made it possible to utilize molecular genetic information to improve the precise diagnosis of psoriasis. In our study, we used single-cell RNA sequencing technology and hdWGCNA to identify psoriasis-specific CD8^+^ T cell subpopulations and screened psoriasis-specific CD8^+^ T cell signature genes, which may be used in the future to assist in the diagnosis of psoriasis and provide a theoretical basis for an in-depth understanding of the immune microenvironment of psoriasis.

CD8^+^ T cells play a crucial role in autoimmune diseases, anti-tumor immunity and anti-infection immunity. Previous researchs have revealed that CD8^+^ T cells infiltrate the skin lesions and blood of psoriasis patients and CD8^+^ T cells can produce IFN-γ, IL-17 and IL-22 (Hijnen et al., 2013), suggesting that CD8^+^ T cells play an important role in the development of psoriasis, but their role in psoriasis has not yet been fully elucidated, and very few studies have explored the specific subtypes and corresponding functions of CD8^+^ T cells. We identified a distinct subset of CD8^+^ T cells based on single-cell RNA sequencing, termed the psoriasis-associated CD8^+^ T cell subset, which is highly infiltrated in psoriatic lesions. We identified GZMB, GNAS, GBP5, FOXP3, LSP1 and CD81 as characteristic genes of psoriasis-associated CD8^+^ T cells, among which, Granzyme B (GzmB), a serine protease, is produced by natural killer (NK) cells and CD8^+^ T cells, and is an vital mediator of skin injury, inflammation and repair (Turner et al., 2019). Studies have shown that the expression of GZMB is elevated in skin lesions and plasma of psoriasis patients (Yawalkar et al., 2001; Kamata et al., 2016), suggesting that GzmB may be involved in CD8^+^ T cell-mediated cell damage and inflammation. The Gαs protein encoded by GNAS gene is a G protein-coupled receptor, which is involved in various signaling pathways, including cell growth, differentiation and apoptosis. The role of GNAS in psoriasis remains unclear. We speculate that GNAS may indirectly affect the development of psoriasis due to its role in immune response and cell proliferation. Guanylate binding protein 5 (GBP5), a member of the guanosine triphosphatase family, is involved in immune and inflammatory responses, and its expression is induced by type I and type II interferons (Kutsch and Coers, 2021), suggesting a potential role for the GBP5 in inflammatory skin diseases. We speculate that GBP5 may be involved in the pathogenesis of psoriasis by regulating inflammatory signaling pathways, such as IFN-γ signaling pathway. Forkhead box protein-3 (FOXP3) is a marker of regulatory T cells (Tregs), especially CD4^+^ Tregs, however, FOXP3 is also expressed in CD8^+^ T cells (Niederlova et al., 2021) and FOXP3 expression is increased in psoriasis lesions (Fujimura et al., 2008). Our results suggest that the high expression of GZBM, GBP5 and FOXP3 in CD8^+^ T cells drives the pathogenesis of psoriasis, therefore, inhibiting the expression of GZBM, GBP5 and FOXP3 is expected to be a new strategy to alleviate the progression of psoriasis. Lymphocyte Specific Protein 1 (LSP1) is mainly involved in cytoskeleton construction and cell migration, so we speculated that LSP1 may affect the migration and localization of inflammatory cells such as T cells and indirectly participate in the pathological process of psoriasis. CD81 is a transmembrane protein that can affect the proliferation and activation of a variety of immune cells. CD81 is highly expressed in both normal and psoriatic tissues, but when CD81 expression is excessively elevated, it promotes the development of psoriasis, which we hypothesize may be related to CD81-mediated hyper- or over-immunization, and this suggests that the immunohyperplasia of CD81 in CD8^+^ T cell subsets may mediate the occurrence of psoriasis, providing new clues for targeted therapy of psoriasis.

Currently, the diagnosis of psoriasis is primarily based on skin lesion characteristics and skin imaging, and there are fewer studies on molecular markers for the early identification of psoriasis patients. In recent years, machine learning has shown great potential in clinical image recognition and disease classification (Anwar et al., 2018), and in the field of dermatology, machine learning algorithms have been used in a variety of diseases such as melanoma, basal cell carcinoma, and onychomycosis, etc. (Haenssle et al., 2018; Harangi, 2018; Marchetti et al., 2018; Zhang et al., 2018), which makes it possible to assist in the diagnosis of diseases by using machine learning methods, of which the first results have been seen in a number of aspects of psoriasis diagnosis, treatment and management (Patrick et al., 2018; Yu et al., 2020; Hammad et al., 2023). Verma et al. (2019) used a machine learning algorithm to predict skin diseases such as psoriasis and achieved a maximum accuracy of 98.64%. Kim et al. (2019) applied machine learning to spectral classification and diagnosis of psoriasis, demonstrating 95% accuracy, 95% sensitivity, and 100% specificity. Dash et al. (2019) constructed a Deep Convolutional Neural Network (DCNN) model to segment psoriasis lesions to assist in the diagnosis of psoriasis. The accuracy of the model was 94.8%, the sensitivity was 89.6%, and the specificity was 97.6%. Due to the diversity and large differences of psoriasis skin lesions, our study obtains six characteristic genes through three machine learning algorithms to predict the occurrence of psoriasis and assist clinical diagnosis and treatment, and shows 100% accuracy, which is higher than previous machine learning image recognition and other methods, and is more conducive to the disease management of psoriasis.

In conclusion, our study identified a psoriasis-specific CD8^+^ T cell subset and its characteristic genes, and established a machine-learning prediction model that provides new ideas for future psoriasis diagnosis. This study is unique in that it delves into the pathogenesis of psoriasis at the single-cell level, identifying a new cell subset specific to psoriasis. Through advanced bioinformatics methods and machine learning/deep learning techniques, it provides new big data-driven tools for the diagnosis of psoriasis, as well as potential new targets for clinical treatment.

## Data Availability

The data presented in the study are deposited in the Gene Expression Omnibus (GEO) repository, accession number GSE151177 and GSE54456.
